# Dynamic calibration of higher eigenmode parameters of a cantilever in atomic force microscopy by using tip–surface interactions

**DOI:** 10.3762/bjnano.5.200

**Published:** 2014-10-29

**Authors:** Stanislav S Borysov, Daniel Forchheimer, David B Haviland

**Affiliations:** 1 Nanostructure Physics, KTH Royal Institute of Technology, Roslagstullsbacken 21, SE-106 91 Stockholm, Sweden; 2 Nordita, KTH Royal Institute of Technology and Stockholm University, Roslagstullsbacken 23, SE-106 91 Stockholm, Sweden; 3 Theoretical Division, Los Alamos National Laboratory, Los Alamos, NM 87545, USA

**Keywords:** atomic force microscopy, calibration, multimodal AFM, multifrequency AFM

## Abstract

We present a theoretical framework for the dynamic calibration of the higher eigenmode parameters (stiffness and optical lever inverse responsivity) of a cantilever. The method is based on the tip–surface force reconstruction technique and does not require any prior knowledge of the eigenmode shape or the particular form of the tip–surface interaction. The calibration method proposed requires a single-point force measurement by using a multimodal drive and its accuracy is independent of the unknown physical amplitude of a higher eigenmode.

## Introduction

Atomic force microscopy [1] (AFM) is one of the primary methods of surface analysis with resolution at the nanometer scale. In a conventional AFM an object is scanned by using a microcantilever with a sharp tip at the free end. Measuring cantilever deflections allows not only for the reconstruction of the surface topography but also provides insight into various material properties [2–3]. If deflection is measured near one of the cantilevers resonance frequencies, an enhanced force sensitivity is achieved due to multiplication by the sharply peaked cantilever transfer function. Measurement of response at multiple eigenmodes can provide additional information about the tip–surface interactions [4–11].

**Figure 1:**
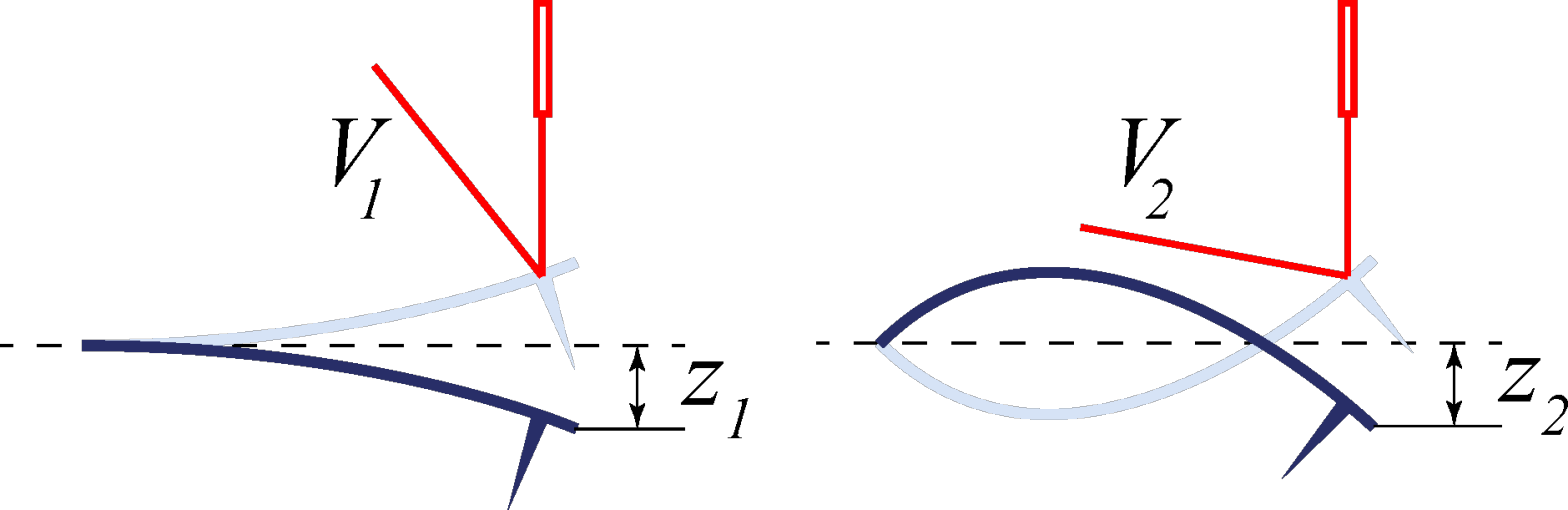
Schematic illustration of the two first flexural eigenmode shapes of a rectangular cantilever and an optical detection system. Measuring of the slope at the free end leads to the situation when the equal vertical tip deflections, *z*_1_ = *z*_2_, result in the different detected voltages, *V*_1_ ≠ *V*_2_. In the case of small deflections, *z_n_* is proportional to *V_n_* with some coefficient *α_n_* called optical lever inverse responsivity.

The optical detection system [12] common to most AFM systems leverages a laser beam reflected from the cantilever, measuring the slope rather than its vertical deflection. This underlying principle leads to the measured voltage at the detector being dependent on the geometric shape of the excited eigenmode (Figure 1). While determination of the stiffness and optical lever inverse resposivity (inverse magnitude of the response function of the optical lever [m/V], also known as “inverse optical lever sensitivity”) of the first flexural eigenmode can be performed with high accuracy using a few well-developed techniques [13–21], calibration of the higher eigenmode parameters is still a challenging task. The main problem with the existing theoretical approaches based on the calculation of eigenmode shapes [18,22] is that real cantilevers differ form the underlying solid body mechanical models due to the tip mass [23–24], fabrication inhomogeneities and defects [25–26]. In this paper, we propose a method which overcomes these deficiencies.

The method uses the fact that the tip–surface force is equally applied to all eigenmodes. This approximation is suitable unless the characteristic spatial wave length of an eigenmode shape is significantly bigger than the tip–cantilever contact area. Any other force acting on the whole cantilever, e.g., of thermal or electromagnetic nature, should be convoluted with the eigenmode shape, leading to a different definition of the effective dynamic stiffness. Thus, knowledge of the geometry of cantilever is not required to reconstruct the tip–surface force. The framework proposed harnesses a force reconstruction technique inspired by the Intermodulation AFM [27] (ImAFM), which was recently generalized to the multimodal case [28]. It is worth noting that the proposed calibration method is similar to that described in [29], in which stiffness of the second eigenmode is experimentally defined by using consecutive measurements of the frequency shift caused by the tip–surface interaction for different eigenmodes. In contrast, we propose a simultaneous one-point measurement by using a multimodal drive that avoids issues related to the thermal drift [30] and exploits nonlinearities for higher calibration precision.

## Results and Discussion

### Cantilever model

We consider a point-mass approximation of a cantilever derived from the eigenmode decomposition of its continuum mechanical model, e.g., the Euler–Bernoulli beam theory. Such a reduced system of coupled harmonic oscillators in the Fourier domain has the following form

[1]
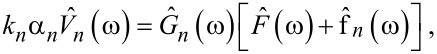


where the caret denotes the Fourier transform, *ω* is the frequency, *k_n_* is the effective dynamic stiffness of the *n*th eigenmode (*n* = 1, … , *N*), *α_n_* is the optical lever inverse responsivity, *V_n_* is the measured voltage (corresponding to the eigencoordinate *z_n_* = *α_n_V_n_*, where total tip deflection is 
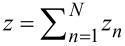
),

[2]
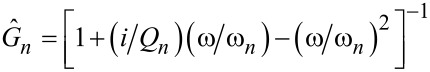


is the linear transfer function of a harmonic oscillator with the resonant frequency *ω_n_* and quality factor *Q_n_*, *F* is a nonlinear tip–surface force and f*_n_* is a drive force. The stiffness is deliberately excluded from the expression for the *G_n_* since the parameters *Q_n_* and *ω_n_* can be found by employing the thermal calibration method [14,17]. Note that if the force amplitudes on the right hand side of Equation 1 are known, one immediately gets *k_n_* and *α_n_* by taking the absolute values in combination with the equipartition theorem

[3]
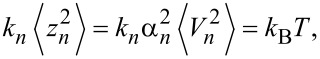


where 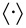 is a statistical average, *k*_B_ is the Boltzmann constant and *T* is an equilibrium temperature.

### Spectral fitting method

The task at hand requires reconstruction of the forces on the right hand side of Equation 1 from the measured motion spectrum. Firstly, it is possible to remove the unknown drive contribution, 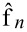, for each *n*, by means of subtraction of the free oscillations spectrum, 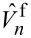 (far from the surface, where *F* ≡ 0), from the spectrum of the engaged tip motion, 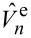 (near the surface). It gives the following relationships

[4]
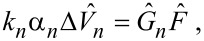


where 
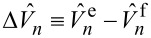
. For the high-*Q* cantilevers, the measured response near each resonance 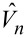 may be separately detected with the high signal-to-noise ratio (SNR). Neglecting possible surface memory effects, *F* depends on the tip position *z* and its velocity 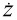 only. With this assumption, the force model to be reconstructed has some generic form

[5]
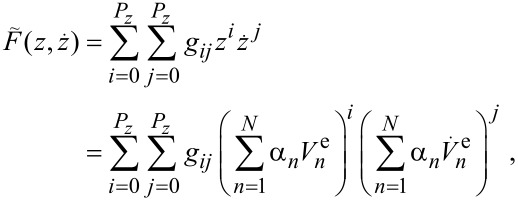


with 
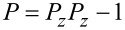
 unknown parameters *g_ij_* (*g*_00_ is excluded because it corresponds to the static force) which can be found by using the spectral fitting method [31–32]: Substitution of Equation 5 in Equation 4 yields a system of linear equations for *g_ij_*. However, this system becomes nonlinear with respect to the unknown *k_n_* and *α_n_*.

### Intermodulation AFM

Assuming that *α*_1_ and *k*_1_ are calibrated by using one of the methods mentioned in the Introduction, the resulting system contains 2(*N* − 1) + *P* unknown variables. Use of the equipartition theorem (Equation 3) for each eigenmode gives us *N* − 1 equations and the remaining equations should be defined by using Equation 4 for the known response components in the motion spectrum. If the force acting on a tip over its motion domain is approximately linear (*P* = 1), one drive tone at each resonant frequency is enough to determine the system. However, when the force behaves in a nonlinear way (*P* > 1), as is usually the case, more measurable response components in the frequency domain are needed. The core idea of ImAFM relies on the ability of a nonlinear force to create intermodulation of discrete drive tones in a frequency comb. Driving an eigenmode subject to a nonlinear force on at least two frequencies 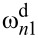 and 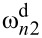 gives a response in the frequency domain not only at these drive frequencies and their higher harmonics but also at their linear combinations 
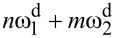
 (*n* and *m* are integers) called intermodulation products (IMPs). Use of the small base frequency 
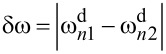
 results in the concentration of IMPs close to the resonance, which opens the possibility for their detection with high SNR. This additional information can be used in Equation 4 for the reconstruction of nonlinear conservative and dissipative forces [28,31–33] with the only restriction that IMPs in the different narrow bands near resonances contain the same information about the unknown force parameters [28].

### Calculation details

In the rest of the paper, we consider a bimodal case implying straightforward generalization for *N* > 2 eigenmodes. Equation 1 is integrated by using CVODE [34] for two different sets of cantilever parameters from Table 1. The cantilever is excited by using multifrequency drive (specified below) with frequencies being integer multiples of the base frequency *δω* = 2*π*·0.1 kHz. The tip–surface force *F* is represented by the vdW-DMT model [35] with the nonlinear damping term being exponentially dependent on the tip position [36]

**Figure 2:**
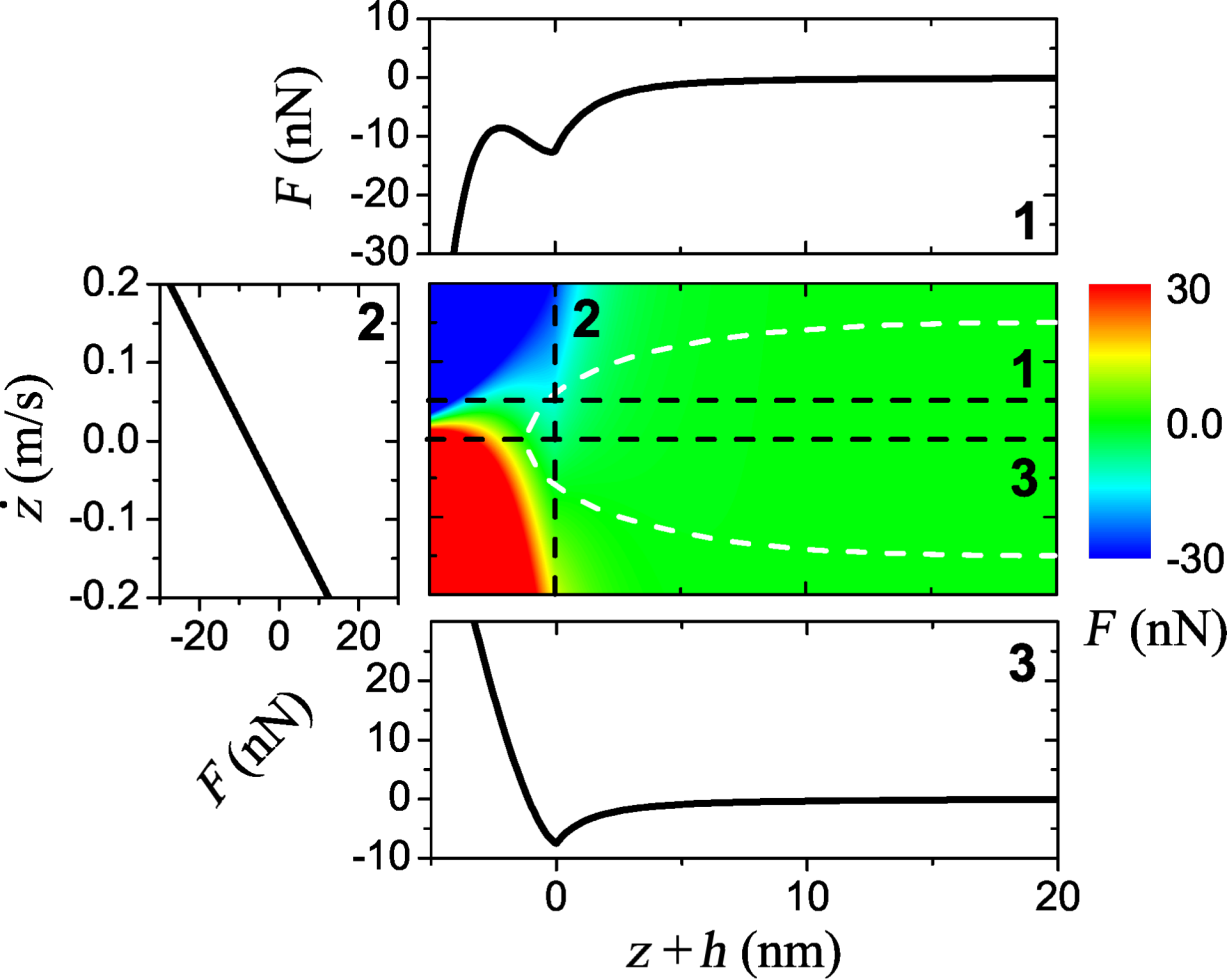
The tip–surface force (Equation 6) used in the simulations. The white dashed line corresponds to a phase space trajectory of the bimodal stiff cantilever with the eigenmode amplitudes *A*_1_ = *A*_2_ = 12.5 nm and reference height *h* = 17 nm. Cross-sections for different values of *z* and 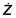 are shown: The projections (1) and (2) correspond to the lines 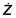 = 0.05 m/s and *z* = 0 nm respectively; the conservative part (3) corresponds to the line 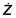 = 0 m/s.

[6]
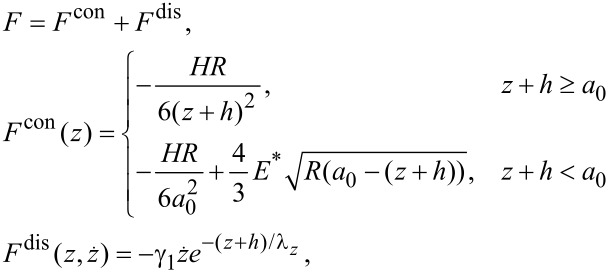


where *h* is a reference height. Its conservative part, *F*^con^, has four phenomenological parameters: the intermolecular distance *a*_0_ = 0.3 nm, the Hamaker constant *H* = 7.1 × 10^−20^ J, the effective modulus E^*^ = 1.0 GPa and the tip radius *R* = 10 nm. The dissipative part, *F*^dis^, depends on the damping factor *γ*_1_ = 2.2 × 10^−7^ kg/s and the damping decay length *λ_z_* = 1.5 nm. The force (Equation 6) and its cross-sections are depicted in Figure 2.

**Table 1:** Cantilever parameters used for the numerical calculations in the paper. Last column *E* is a total oscillation energy of a free cantilever with the equal eigenmode amplitudes *A*_1_ = *A*_2_ = 1 nm.

cantilever	*ω*_1_ ((2*π*)^−1^ kHz)	*ω*_2_/*ω*_1_	*Q*_1_	*Q*_2_/*Q*_1_	*k*_1_ (N/m)	*k*_2_/*k*_1_	*α*_2_/*α*_1_	*E* (fJ)

soft	82.7	6.35	220.0	2.9	5.0	40.0	2.0	1.02
stiff	300.0	6.3	400.0	3.0	40.0	50.0	2.0	0.105

### Calibration by using a nonlinear tip–surface force

In order to find *k*_2_ and *α*_2_ from the nonlinear system (Equation 3 and Equation 4), we first solve the linear system for the force parameters *g_ij_*. It is then convenient to compare only the conservative part of the tip–surface force given its non-monotonic behavior. There are two methods to require equality of the reconstructed forces 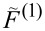 (using the band near the first eigenmode) and 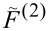 (near the second eigenmode). The first method is to check the difference between the corresponding parameters 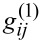 and 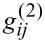. However, this approach is not suitable because two completely different sets of coefficients might define very similar functions on the interval of the actual engaged tip motion, [*A*^min,e^; *A*^max,e^], where *A*^max^ = max *A*(*t*) = max *z*(*t*). As numerical simulations have shown, the error function does not have a well-defined global minimum and it is highly sensitive to reconstruction errors. An alternative approach is to minimize a mean square error function in real space

[7]
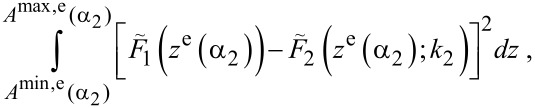


which in most regimes of the tip motion has only one global minimum lying in the deep valley defined by the curve 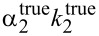. Moreover, increasing the reconstructed polynomial power, *P_z_*, makes this valley deeper and hence more resistant to noise. This method allows for the estimation of the product *α*_2_*k*_2_ with higher accuracy than α_2_ and *k*_2_ separately.

**Figure 3:**
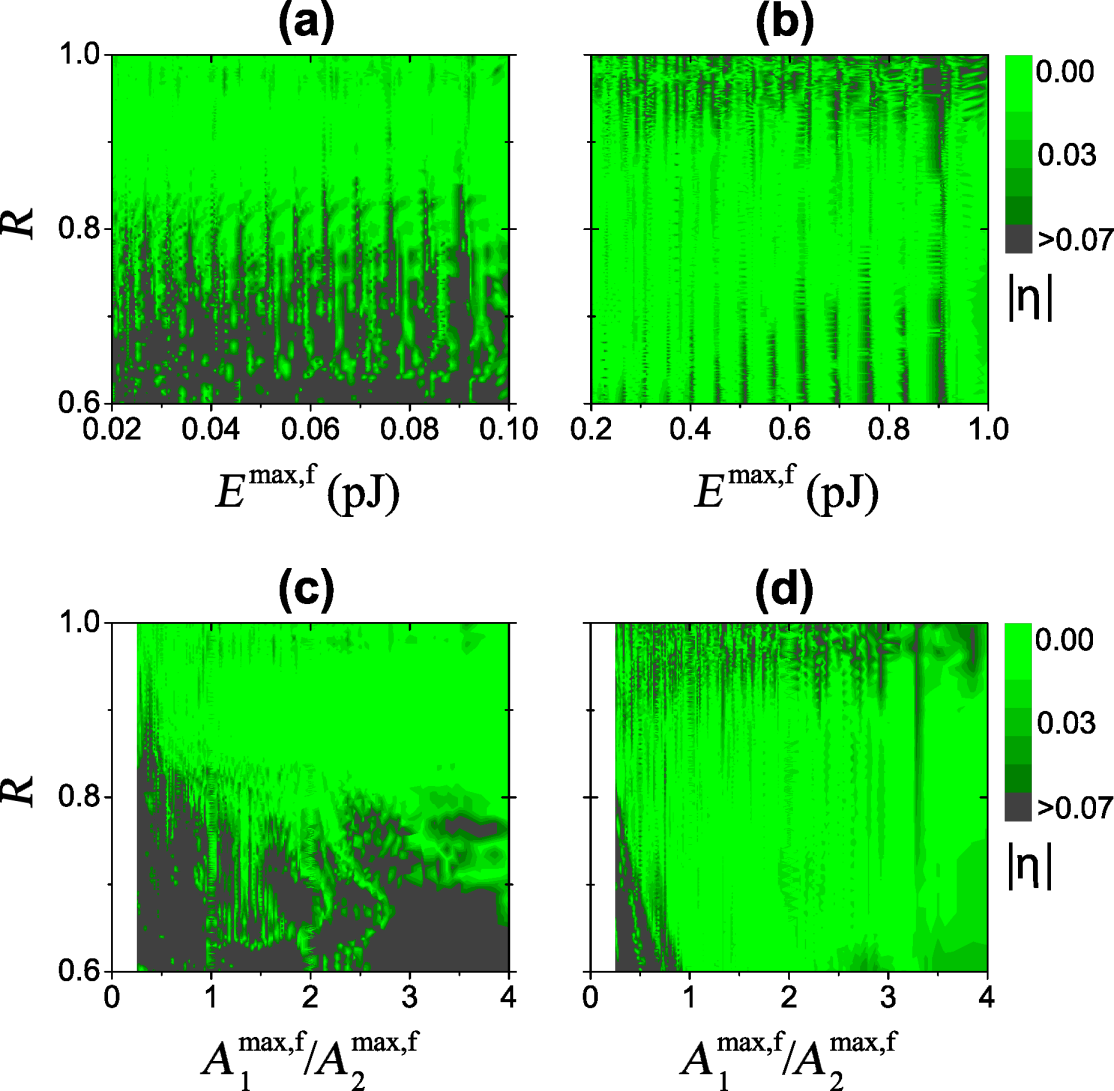
Absolute value of the relative calibration error *η* of *k*_2_*α*_2_ as a function of the ratio *R* = *h*/*A*^max,f^, total maximum free oscillation energy *E*^max,f^ (top row) and the ratio of maximum free amplitudes 
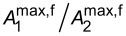
 (bottom row) for the soft (a), (c) and stiff (b), (d) cantilever, respectively.

Figure 3 shows the absolute value of the relative error

[8]
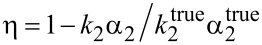


plotted in the plane of maximum free oscillation energy 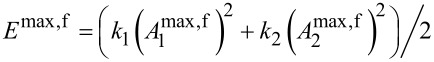 and the ratio *R* = *h*/*A*^max,f^. The relative calibration error is small over a wide range of oscillation energy and probe height for both soft (Figure 3a) and stiff (Figure 3b) cantilevers. The regions of lower error correspond to a large value of the ratio 
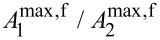
 (Figure 3c and Figure 3d). Experimentally, one can check the stability of calibration by comparing different probe heights and oscillation energies. Finally, the stiff cantilever has a wider region of low error because a higher oscillation energy effectively weakens the nonlinearity.

### Calibration by using a linear tip–surface force

When the interval of the engaged tip motion is small, the tip–surface force (Equation 5) can be linearized. In this case, it is possible to obtain the explicit expression for the stiffness by using a linear model 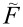 with one unknown parameter *g*_10_

[9]
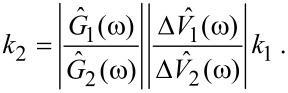


**Figure 4:**
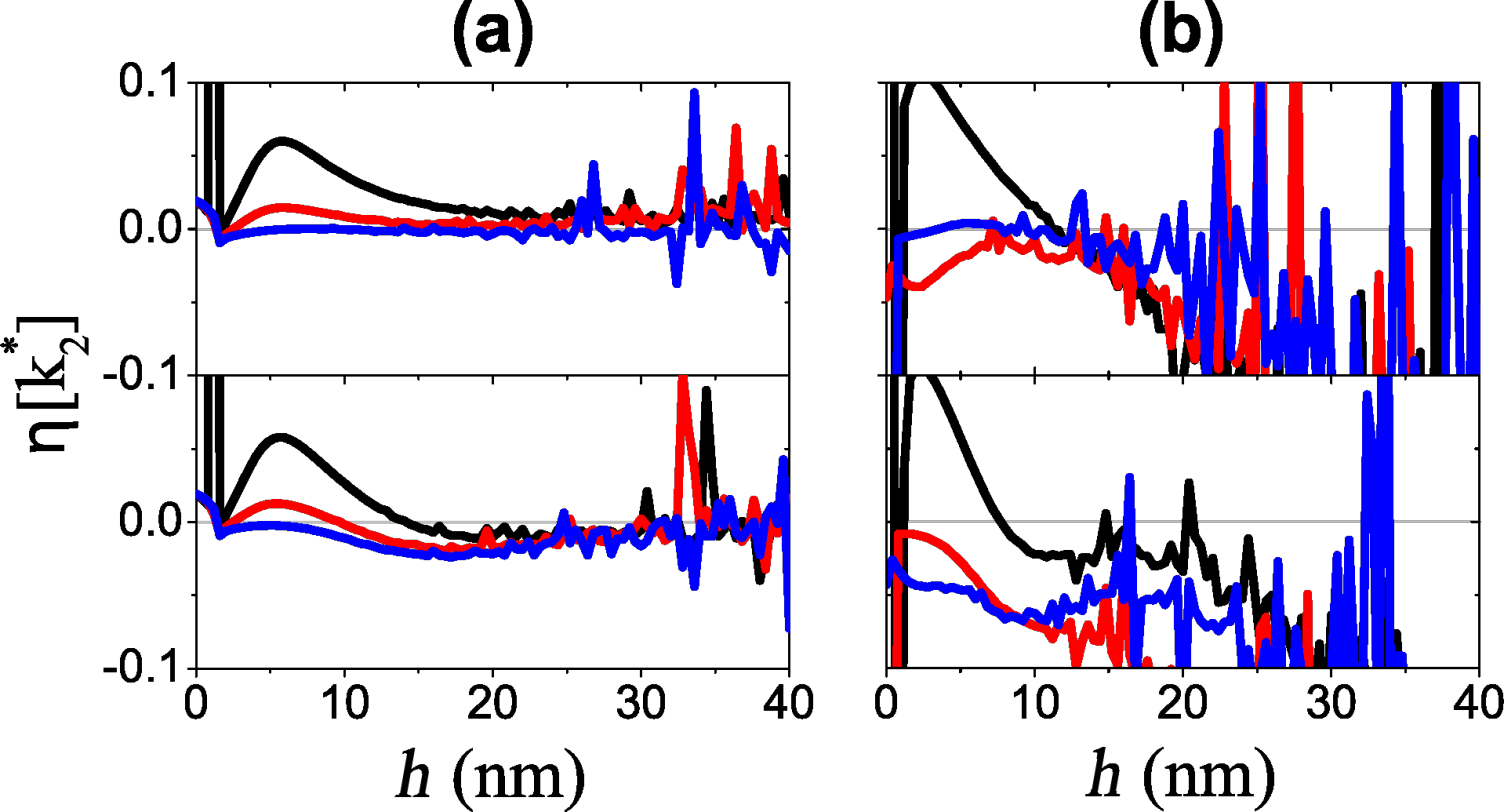
Relative calibration error of the calibrated stiffness *k*_2_, 
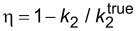
, using Equation 9 for two different cantilevers: (a) soft and (b) stiff, with different free eigenmode oscillations amplitudes: 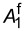 = 1 nm (top), 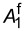 = 3 nm (bottom), 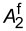 = 0.1 nm (blue), 1.0 nm (red) and 2.0 nm (black).

If *g*_01_ is used instead, *k*_2_ should be additionally multiplied by *ω*_1_/*ω*_2_. As previously mentioned, the multimodal drive at the resonant frequencies *ω*_1_ and *ω*_2_ (more precisely, their discrete approximations defined by *δω*) produces enough response components to find *k*_2_. The corresponding domain of the engaged tip motion and eigenmode sensitivity to the force are defined by the energy scale factor 
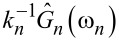
. Therefore, calibration of the softer cantilever can be performed with higher accuracy, while for the stiff cantilever, small drive amplitudes are required for acceptable calibration results (Figure 4). Near the surface, the force is highly nonlinear, making the tip prone to sudden jumps to the contact. From an experimental point of view, probing only the attractive part of the interaction with small oscillation amplitudes protects the tip from possible damage since the dissipation is almost zero in this regime.

Finally, the linear method is dependent on the unknown higher eigenmode free amplitude, 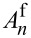, which must be small for the linear approximation to be valid. Since 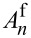 is not known a priori, one can use the following formula to try to make a rough guess given the known free amplitude of the first mode for the particular drive voltage amplitude

[10]
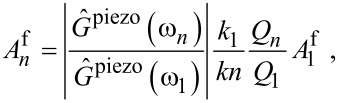


where 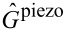 is a transfer function of the piezoelectric shaker.

### Implementation

Summarizing the ideas presented, an experimental implementation of the proposed calibration would consist of the following steps, with related sources of possible calibration error:

1. *Construct a multimode drive and measure the free motion spectrum of a cantilever*, *V*^f^. Since the free motion components can be used instead of the drive force, the real physical amplitude of the cantilever and the transfer function of the piezo shaker do not contribute to the accuracy of the method. However, as numerical calculations have shown, the method is sensitive to SNR of the measurement, performing poorly when the SNR is too small. Therefore, a drive with approximately the same SNR for all eigenmodes would be a good option.
2. *Move the cantilever closer to the surface and measure its engaged motion spectrum*, *V*^e^. In principle only one measured spectrum corresponding to a particular probe height is enough for calibration purposes. However, the use of spectra at different probe heights will improve calibration precision. The method may be applied to both soft and stiff cantilevers, but works best when nonlinearities are weak. Thus the amplitude contraction of the engaged cantilever oscillations with respect to the free motion should be about 10–20%.
3. *Choose a particular model for the tip*–*surface interaction and solve nonlinear system* Equation 4 *for unknown parameters using the measured difference spectrum V*^e^ − *V*^f^. If the exact expression for *F* is unknown, ImAFM provides enough information to reconstruct it in a generic form, e.g., as power series (Equation 5). As numerical simulations have shown, a more realistic model gives better calibration with the same error function (Equation 7). Making use of any additional prior information about the cantilever also improves the accuracy of the calibration. For instance, *ω_n_*, *Q_n_* and 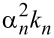 can be estimated by using the thermal calibration method [14,17] and the equipartition theorem (Equation 3).

Theoretically, the method should work in liquid or high-damping environments, however, experimental implementation in liquid will suffer from actuation-related effects, squeeze-film damping close to the surface and spurious resonances. [37].

## Conclusion

We outlined a theoretical framework for experimental calibration of cantilever parameters by using the tip–surface force with one-point measurement and a multimodal drive. The proposed approach does not require any knowledge of the geometry of the cantilever or the form of the tip–surface interaction. The method possesses a high calibration accuracy independent of the a priori unknown amplitude of the higher eigenmode.
